# Spectral‐Kinetic Synergy in Au‐Network Engineered FeTiO_3_: A Multi‐Field Coupling Strategy for Lunar In Situ Resource Utilization

**DOI:** 10.1002/advs.76857

**Published:** 2026-08-06

**Authors:** Yahang Wang, Quanxin Wang, Pakkin Leong, Shi Feng, Meng Wang, Runyang Mo, Xianjin Shi, Xiaohui Li, Gangqiang Zhu, Chipui Tang

**Affiliations:** ^1^ State Key Laboratory of Lunar and Planetary Sciences Macau University of Science and Technology Taipa Macao People's Republic of China; ^2^ School of Physics and Information Technology Shaanxi Normal University Xi'an People's Republic of China; ^3^ State Key Lab of Loess and Quaternary Geology (SKLLQG) Institute of Earth Environment Chinese Academy of Sciences Xi'an People's Republic of China; ^4^ Faculty of Innovation Engineering Macau University of Science and Technology Taipa Macao People's Republic of China

**Keywords:** ilmenite (FeTiO_3_), localized surface plasmon resonance (LSPR), lunar in situ resource, multi‐field coupling

## Abstract

Currently, in situ lunar resource utilization faces two major challenges: traditional photocatalysts cannot effectively absorb and utilize infrared spectra, and there is severe kinetic inhibition. To address this challenge, this paper proposes a pioneering ‘spectral‐kinetic synergy’ strategy, constructing a unique Au‐network engineered ilmenite (AuL‐FeTiO_3_) architecture. This network topology transforms the catalyst into a “photo‐thermal‐electric” multi‐field coupling platform. The reticulated Au architecture functions as a dual‐mode amplifier: it generates a local electromagnetic field orders of magnitude stronger than that of conventional nanoparticles via Localized Surface Plasmon Resonance (LSPR), driving the production of energetic hot electrons; simultaneously, it efficiently harvests broadband infrared light to establish a localized thermal field. Crucially, in situ characterization enables us to visualize and decouple the distinct dynamics of thermally driven lattice electrons vs. plasmonic hot electrons. The study revealed that the local thermal field acts as a “kinetic promoter,” facilitating the injection of a large number of LSPR‐derived hot electrons into the Au‐FeTiO_3_ interface. This mechanism significantly extends carrier lifetime and accelerates interfacial charge transfer kinetics. Crucially, this structure drives efficient photothermal CO_2_ reduction. This work not only elucidates the collaborative mechanism of spectral‐kinetic coupling but also provides a transformative blueprint for designing high‐performance catalysts using indigenous lunar materials.

## Graphical abstract

How can humanity achieve lunar self‐sufficiency? We utilize Au topology networks to modify the abundant lunar ilmenite. This catalyst, under sunlight, can convert waste carbon dioxide into space fuel. This design leverages the moon's natural vacuum for green manufacturing, avoiding the waste of gold and serving as “artificial chloroplasts” to sustain future closed‐loop extraterrestrial ecosystems.

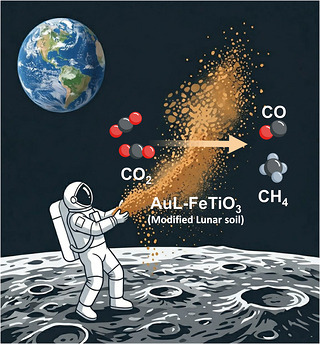

## 1 Introduction

Utilizing lunar in situ resources is crucial for establishing a lunar base and further exploring it. As lunar exploration deepens, effectively utilizing lunar resources has become a crucial issue in promoting lunar base construction and deep space exploration. Photothermal CO_2_ reduction technology is considered a promising solution for converting lunar in situ resources, such as CO_2_ and water ice, into laboratory‐grade raw materials, supporting future scientific experiments and long‐term human presence [1]. FeTiO_3_ in lunar regolith has been identified as a promising catalyst for subsequent in situ resource utilization (ISRU). However, several key challenges remain: unclear carrier dynamics after the introduction of LSPR, low visible light utilization, and a shortage of reactive sites [2, 3]. In particular, the limited response of most catalysts in the visible and near‐infrared wavelengths results in low solar energy utilization, severely limiting the potential for lunar applications [2, 4].

FeTiO_3_ has a perovskite structure. Due to the inherent anisotropy of its crystal structure, effective control of the termination faces is often difficult during synthesis, resulting in low end‐overlayer selectivity and posing challenges for catalyst microstructural design [5, 6, 7, 8, 9]. Studies have found that exposing only a single overlayer agent in perovskites has the advantages of optimizing reactant activation, carrier mobility, and acid/base catalytic properties [3]. Dong et al. achieved p‐type surface doping by end‐overlayer MAPbI_3_ with MAI, promoting carrier transport and exhibiting excellent photocatalytic performance [10]. Similar SrTiO_3_ surface terminations have also been used to modulate acid‐base catalytic properties, enabling the dehydrogenation/dehydration of propanol to produce different products [3]. Considering these advances, it is speculated that other materials can be used for end‐overlayer in addition to in situ overlayer. et al. used carbon coatings to overlaid perovskites in tandem cells, achieving unbiased CO_2_ reduction, suggesting that other materials can also serve as overlayer agents. Furthermore, conventional noble metal‐loaded nanoparticles or nanorods are typically uniformly loaded on the catalyst surface, which can result in low solar light utilization and weaken the LSPR effect. Numerous studies have shown that the morphology and arrangement of the loaded metal significantly influence the surface LSPR intensity [11, 12, 13, 14, 15, 16]. Tajei et al. studied hat‐shaped Au and found that it significantly broadened the infrared absorption band [17]. They also studied Au nanoraspberry arrays and found that their LSPR effect was two times better than that of Au nanoparticle arrays [16]. This superiority was attributed to the small‐scale protrusions on the nanoraspberry surface, which enhanced the local electric field and sensing performance. Interestingly, Feng et al. manipulated the roughness of Au nanoparticles by using thiol ligands as morphology‐directing agents. Increased surface roughness simultaneously enhanced the LSPR response range and surface‐enhanced Raman spectroscopy intensity [17]. The LSPR effect is also a hot topic in photocatalysis. Li et al. investigated the LSPR effect of Ag dendrites and found that they achieved a broad light absorption band and carrier separation efficiency, and exhibited excellent degradation performance. The structure‐activity relationship of the LSPR effect has also attracted much attention in the CO_2_ reduction reaction. For example, by constructing a g‐C_3_N_4_/Pd/MoO_3‐x_ dual‐plasma system, they exploited the dual LSPR effect to broaden the optical response range, improving photocatalytic activity and near‐infrared performance [18]. Similarly, in the ZnO/Au/g‐C_3_N_4_ ternary composite catalyst, Au nanoparticles not only serve as an electron transfer bridge, but also as an LSPR excitation source to accelerate the separation of electron‐hole pairs, thereby maximizing the electron transfer efficiency [19]. The LSPR effect can also effectively promote charge separation, improve the adsorption and activation efficiency of CO_2_, and further optimize the reaction process [20]. However, in situ end‐overlayer of perovskite overlayer has good research significance in theory, but the improvement effect on actual catalytic performance is still limited. Traditional loaded nanoparticles are usually evenly distributed on the catalyst surface. Although this method can increase the number of adsorption sites, it also limits the effective use of sunlight, and the carrier dynamics behavior brought about by the LSPR effect is not clear [21, 22].

Therefore, to address the critical challenges of cryogenic lunar surface temperatures and inefficient solar spectrum utilization, this work proposes an innovative ‘Au‐network engineering’ strategy, constructing a unique Au‐overlaid FeTiO_3_ architecture. The unique reticulated Au overlayer successfully transforms infrared light into localized heat and high‐energy hot electrons, establishing a robust spectral‐kinetic synergy. In this coupled system, the thermal field functions as a kinetic promoter to lower the reaction energy barrier, while the localized electromagnetic field drives the generation and injection of energetic electrons. This synergy not only overcomes the efficiency limitations of a single energy mode, but the optimized electronic structure at the interface also significantly extends carrier lifetime, thereby synergistically optimizing the dynamics of the entire process from photothermal conversion and charge separation to surface reactions. This work provides a highly stable and feasible path to overcome the bottleneck of efficient CO_2_ conversion in lunar environments.

## 2 Results

### 2.1 Catalyst Structure

**FIGURE 1:**
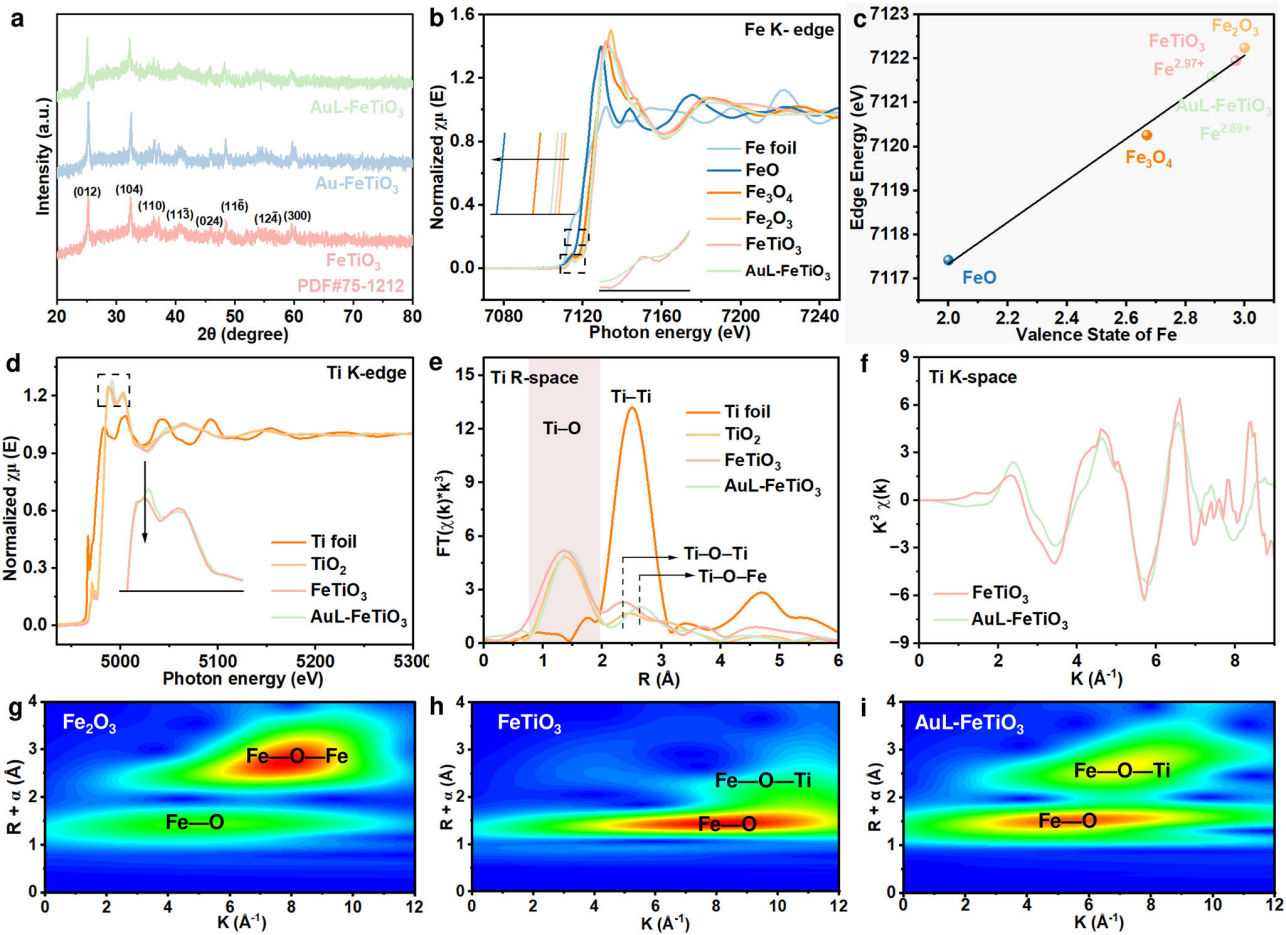
Structural Characterization. (a) XRD spectra of FeTiO_3_, Au‐FeTiO_3_, and AuL‐FeTiO_3_. (b) Fe K‐edge XANES spectra, with the insets showing the front and edge peaks. (c) Fe oxidation states in different samples calculated using Fe K‐edge XAFS. (d) Ti K‐edge XANES spectra, with the insets showing the white peaks. R‐space (e) and k‐space (f) of Ti. Wavelet transforms of Fe_2_O_3_ (g), FeTiO_3_ (h), and AuL‐FeTiO_3_ (i).

Magnetron sputtering, by virtue of its exceptional process flexibility and broad target compatibility, can directly leverage the inherent high‐vacuum environment of the Moon. Featuring pollution‐free operations and minimal disturbance to the extraterrestrial ecology, it represents a highly promising strategy for lunar in situ surface modification and catalyst construction. Therefore, in this work, an Au network coating was prepared on the FeTiO_3_ surface using sputtering technology. X‐ray diffraction (XRD) patterns and Fourier transform infrared (FTIR) spectra corroborate the preservation of the intrinsic monoclinic crystal structure following the Au deposition (Figure 1a and Figure S1). Consistent with this, surface area and pore size analysis reveal negligible alteration in the textural properties, indicating that the overlayer construction does not compromise the structural integrity of the substrate (Figure S2). Furthermore, x‐ray photoelectron spectroscopy (XPS) was employed to probe the specific surface chemical states of the Au‐network engineered FeTiO_3_ (Figure S3). Unlike the typical metallic state Au^0^ species in conventional chemical reduction methods, the sputtered gold network capping layer exhibits unique cationic characteristics (Au^+^). This difference in valence state is a characteristic of strong interfacial electronic coupling between the Au network and the oxide substrate. Although the chemical environment of Fe and Ti remains largely undisturbed after modification, quantitative analysis shows that the concentration of oxygen vacancies (VOs) in AuL‐FeTiO_3_ is significantly increased compared to conventional Au‐loaded samples. This structural feature is crucial because it is decisive in generating abundant active sites, thereby significantly promoting the adsorption and activation of reactive molecules.

To further investigate the modulation effect of the Au network overlayer on the local atomic structure and electronic configuration of the catalyst, we performed x‐ray absorption fine structure (XAFS) spectroscopy analysis. The Fe K‐edge XANES spectrum (Figure 1b and Figure S4) confirms that the unique Au network did not disrupt the tetrahedral coordination geometry of Fe‐O in the FeTiO_3_ substrate [23]. Notably, a significant redshift occurred at the absorption edge position. Based on the linear fit between the absorption edge energy and oxidation state (Figure 1c), quantitative analysis shows that the average valence state of Fe in AuL‐FeTiO_3_ decreased to +2.89, lower than the +2.97 of pure FeTiO_3_. This decrease in valence state directly suggests the injection of interfacial electrons or the formation of oxygen vacancies. For the Ti K‐edge (Figure 1d), the pre‐peak characteristics indicate that the Ti‐O octahedral framework remained intact after modification. Crucially, AuL‐FeTiO_3_ exhibited a higher white line intensity (corresponding to an increased probability of Ti 1s→4p electron transitions). This reveals that the Au network overlayer induces the generation of abundant coordinatively unsaturated Ti sites. These sites function as critical Lewis acid centers, substantially facilitating the chemisorption of reactant molecules [24, 25]. Further EXAFS analysis (Figure 1e and Figure S5) shows that the local coordination peak positions of Ti in R space remain unchanged, confirming the stability of the first shell. However, in k space (Figure 1f), we observe a significant decrease in oscillation intensity after the introduction of AuL. This does not signify a destruction of the crystal structure, but rather indicates an increase in local structural disorder, i.e., the introduction of abundant defect sites such as oxygen vacancies. Finally, the wavelet transform (WT) spectrum of Fe (Figure 1g and Figure S6) visually resolves the characteristic lobe of Fe‐O‐Ti located at R ≈ 2.5 Å. This feature remains stable before and after modification, indicating that although the Au network overlayer introduces defects and valence state changes, the framework connectivity of FeTiO_3_ remains unaffected, ensuring the structural stability of the catalyst.

### 2.2 Multi‐Field Coupling

**FIGURE 2:**
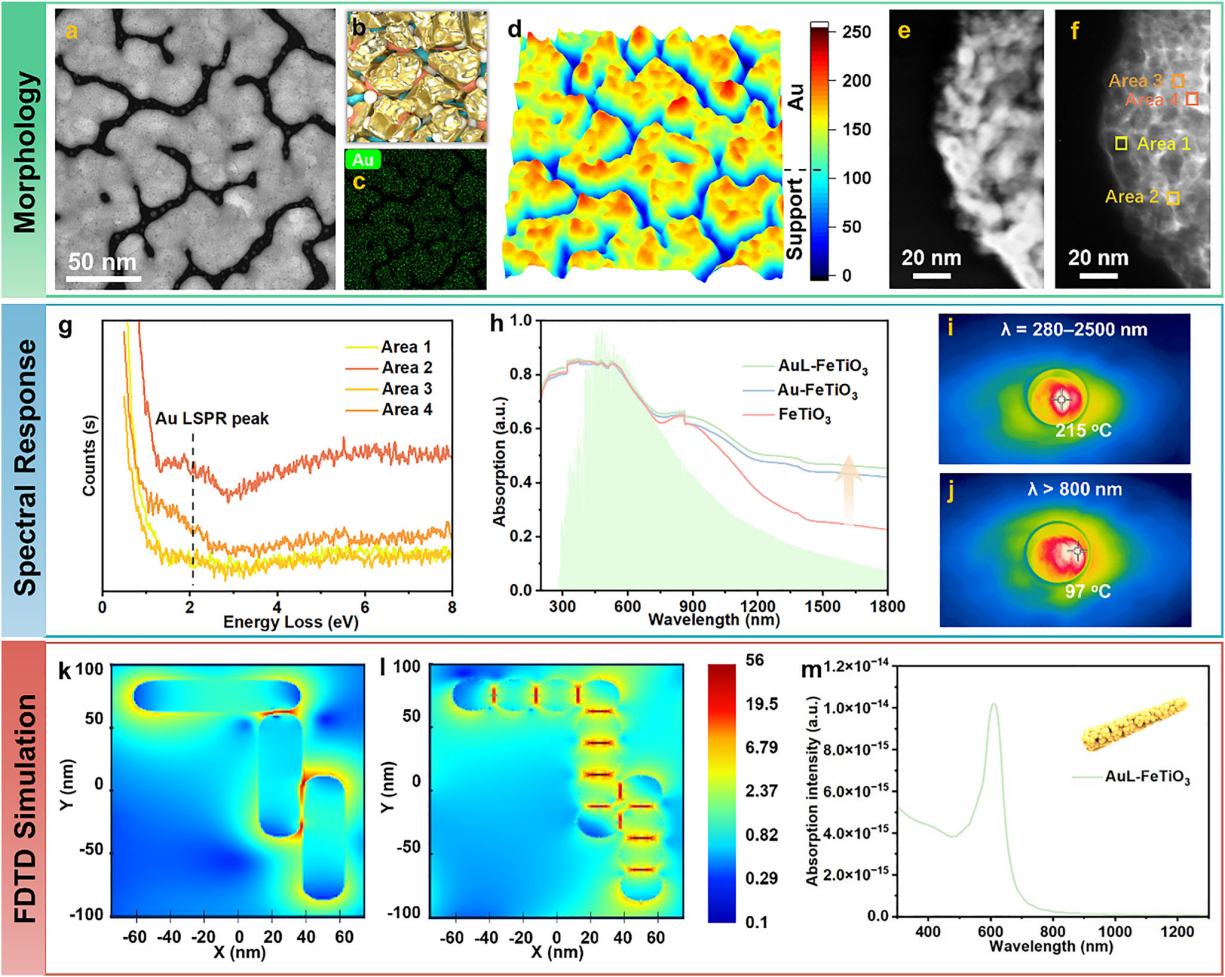
Multi‐field coupling. (a) spherical aberration‐corrected high‐resolution electron microscopy image of AuL‐FeTiO_3_. (b) Schematic diagram of AuL overlayer on the FeTiO_3_ surface. (c) EDS mapping image of the Au overlayer. (d) Three‐dimensional Gaussian function fitting plot. HAADF‐STEM (e) and BF‐STEM (f) images of AuL. (g) Energy Loss Spectrum of AuL‐FeTiO_3_. (h) Optical absorption spectra of FeTiO_3_, Au‐FeTiO_3_, and AuL‐FeTiO_3_. (i,j) Infrared thermal images of AuL‐FeTiO_3_ taken at different wavelengths. (k) FDTD simulation showing enhanced absorption of AuL‐ overlaid nanorods. LSPR simulation of the near‐field electric field intensity of Au nanorods (l) and AuL‐ overlaid nanorods (m).

Scanning electron microscopy (SEM) images revealed the unique network morphology on the AuL‐FeTiO_3_ surface (Figure S7). However, the precise composition and topological hierarchy of this network remained to be unambiguously resolved. To further resolve the composition and fine structure of this complex network at the atomic scale, we employed aberration‐corrected high‐angle annular dark‐field scanning transmission electron microscopy (HAADF‐STEM) (Figure 2a). The HAADF‐STEM imagery provides unambiguous visualization of the Au network structure adhering to the FeTiO_3_ surface, effectively validating our rationalized model of the Au network overlayer (Figure 2b). Elemental map further confirms that the network framework is entirely composed of Au elements (Figure 2c). Combining the TEM images with spectral intensity maps fitted by a three‐dimensional Gaussian function (Figure 2d, Figures S8 and S9), the three‐dimensional morphological features of this structure are revealed: the AuL network overlayer is not a flat film, but a three‐dimensional architecture composed of interconnected gold nanoparticles with a significant height difference from the FeTiO_3_ substrate. As is well known, gold nanostructures exhibit significant LSPR effects [26]. However, most existing research focuses on isolated nanoparticles, with few studies exploring the structure‐property relationship between this unique “network interconnection structure” and its LSPR field strength [27, 28]. Given the extreme challenge of directly experimentally characterizing its complex localized electromagnetic field distribution at the nanoscale, we subsequently employed finite‐difference time‐domain (FDTD) simulations to theoretically visualize and quantify the field enhancement effect of this special structure.

To directly visualize the LSPR modes and spatial distribution of the Au network overlayer at the sub‐nanometer scale, we combined HAADF, bright‐field (BF) imaging, and electron energy loss spectroscopy (EELS). The complementary contrast between the HAADF and BF images sharply delineates the skeletal architecture of the Au network (Figure 2e,f and Figure S10). Selective EELS analysis revealed a striking phenomenon: the AuL‐FeTiO_3_ interface boundary region, which appears as a high‐contrast area in the BF image, exhibits extremely strong plasmon EELS signals (Figure 2g and Figure S10) [29]. This provides direct experimental evidence that the entire Au network overlayer is not a mere physical coating, but rather constitutes a large‐area, continuously distributed plasmonically active network. Compared to isolated gold nanoparticles, this interconnected network introduces high‐density “hot spots,” significantly expanding the effective range of the LSPR field and enhancing the local electromagnetic field intensity. Macroscopic spectral responses further confirm this microscopic mechanism (Figure 2h). Although the characteristic LSPR peak of Au in the visible region (400–500 nm) is partially masked by the interband transition absorption of FeTiO_3_, a significant broadband absorption enhancement was observed in the infrared region. This infrared response is not accidental but is attributed to collective electronic oscillations induced by strong coupling between adjacent nanounits in the Au network architecture. This excellent broadband light‐harvesting capability directly translates into superior photothermal conversion performance. Infrared thermal imaging (Figure 2i,j and Figure S11) confirms that the AuL‐FeTiO_3_ architecture reaches its highest equilibrium temperature under full‐spectrum illumination. This superior thermal performance is attributed to the synergistic effect of LSPR‐induced localized heating and the inherent structure‐derived photothermal effect of the Au network. Furthermore, band structure analysis (Figure S12) shows that the bandgap of FeTiO_3_ remains stable before and after modification, ruling out the contribution of semiconductor band narrowing to the absorption enhancement. In summary, EELS and spectral characterization successfully confirmed that the AuL‐FeTiO_3_ not only constructed a high‐intensity LSPR field, but also achieved efficient broadband photothermal conversion.

To theoretically elucidate the LSPR enhancement mechanism of AuL overlayer compared to conventional Au nanoparticles, we performed FDTD simulations of Au structures with different morphologies and arrangements (Figure 2k,i and Figure S13). For conventional Au nanoparticles, the large interparticle spacing blocks near‐field coupling, and the LSPR electric field is mainly confined to the neighborhood of individual particles. To construct a theoretical model that more closely resembles the real physical picture, based on the structural features of SEM images (Figure S13b, region 1), we rationalized the Au overlayer as an array of interconnected nanorods. Simulation results show that, unlike isolated nanoparticles (Figure S13d), the array structure induces a significant field enhancement effect at the intersection nodes of the nanorods, forming a high‐density plasmon “hot spot” (Figure 2k). Subsequently, combined with the fine lattice information provided by high‐resolution transmission electron microscopy (HR‐TEM) (Figure S13c), we further optimized the simulation model. The optimized results confirm that the AuL‐FeTiO_3_ exhibits the strongest and most spatially distributed LSPR effect (Figure 2l). By virtue of the coherent coupling between adjacent domains within the network topology, the local electromagnetic field intensity realizes an enhancement of several orders of magnitude relative to discrete gold nanoparticles. Subsequent spectral analysis (Figure 2m and Figure S13g,h) demonstrates that this topological superiority not only amplifies absorption in the visible spectrum but, more critically, extends the absorption envelope deep into the infrared regime, exhibiting an enhancement magnitude that far surpasses that of other reference models. The fundamental origin of this superior performance lies in the unique continuous network topology of the Au network overlayer, which facilitates stronger collective oscillations of surface free electrons. In essence, this Au‐network engineering strategy maximizes the surface LSPR effect by constructing abundant plasmonic coupling nodes, thereby laying a solid physical foundation for the efficient harvesting and utilization of infrared light.

### 2.3 Performance and Carrier Behavior

**FIGURE 3:**
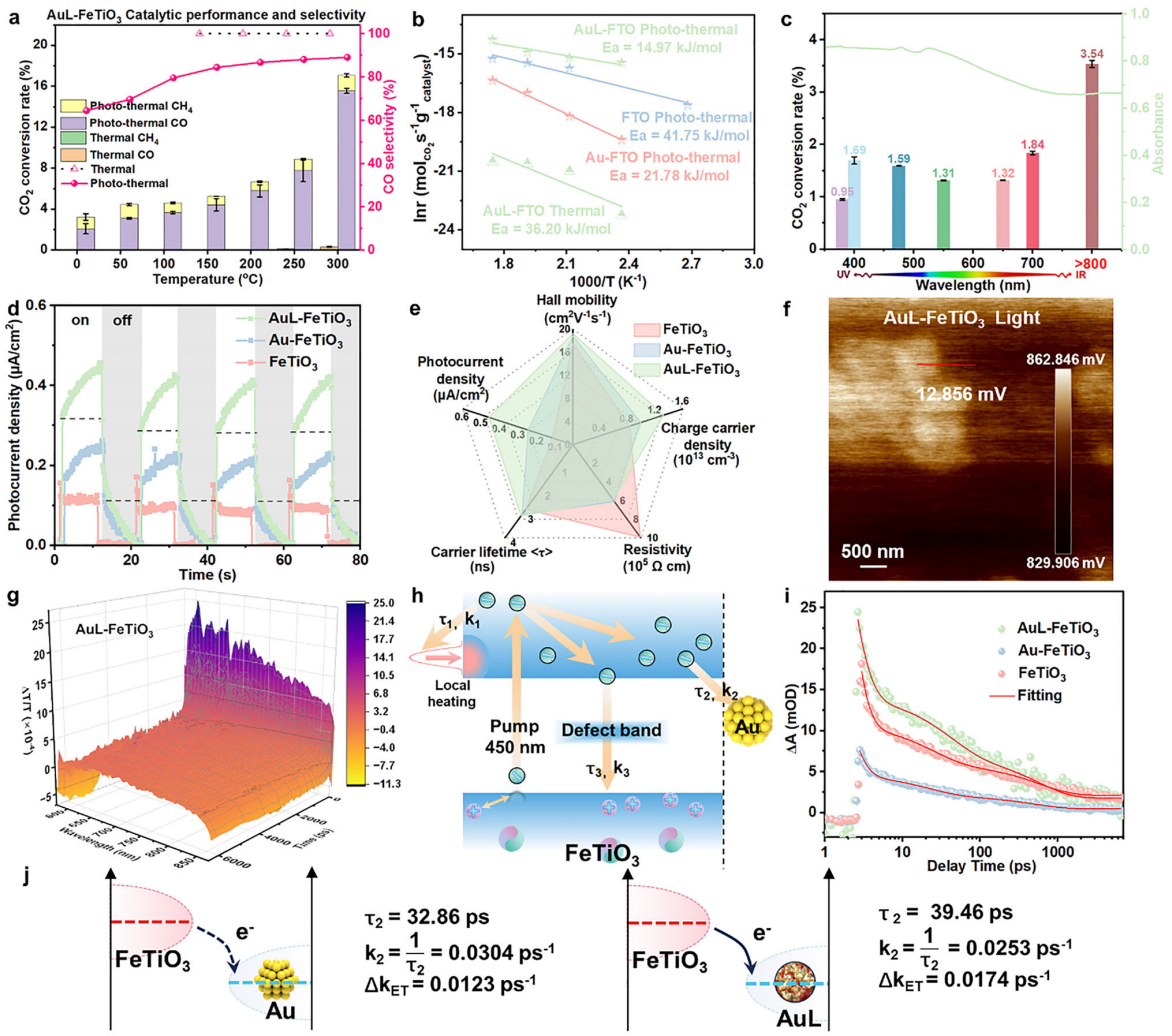
Performance and carrier migration. (a) Photothermal CO_2_ hydrogenation performance of AuL‐FeTiO_3_ and its selectivity for CO (test conditions: 0°C–300°C, 15 mW·cm^−^^2^). (b) Ea activation energy fitting plot. (c) Performance and optical absorption spectra of AuL‐FeTiO_3_ under monochromatic light. (d) Photocurrent diagram. (e) Carrier separation and migration. (f) KPFM image of AuL‐FeTiO_3_ under illumination. fs‐TAs of AuL‐FeTiO_3_ (g) and schematic diagram of different carrier behaviors (h). (i) Exciton relaxation kinetics of FeTiO_3_, Au‐FeTiO_3_, and AuL‐FeTiO_3_. (j) Schematic diagram of charge carrier migration kinetics of Au‐FeTiO_3_ and AuL‐FeTiO_3_.

As shown in Figure 3a and Figure S14, we evaluated the efficiency of the Au network overlayer for the photothermal reduction of CO_2_. The results showed that FeTiO_3_ converted only about 2% of CO_2_ at 300°C, with CO as the product. However, after adding the Au network overlayer, the CO_2_ conversion reached approximately 20%, a tenfold increase compared to FeTiO_3_. It is worth noting that the AuL‐FeTiO_3_ sample also exhibited excellent performance at room temperature, which means that the coupling of LSPR‐induced hot electrons and photothermal effects significantly enhanced the performance. Subsequent long‐term durability tests showed that the AuL‐FeTiO_3_ architecture did not exhibit significant activity degradation over approximately 30 h. (Figures S15 and S16). To quantify the photothermal effect in multi‐field coupling, we used the Ea as an indicator to measure the contribution of the thermal field to the reaction and fitted the Ea of the three samples (Figure 3b). Under photothermal conditions, the activation energy of AuL‐FeTiO_3_ is lower than that under pure thermal conditions, indicating that the photothermal effect reduces the activation energy of the reaction. The photothermal effect of the FeTiO_3_ sample is significantly higher than that of other modification methods. Performance tests of the samples at different wavelengths show that the introduction of the Au network overlayer enhances the infrared performance (Figure 3c). Catalytic evaluation under wavelength‐dependent illumination (Figure 3c) reveals that the sample exhibits superior activity even in the infrared region. This specific IR response is attributed to the efficient broadband photothermal conversion capability enabled by the Au network overlayer. Photocurrent measurements (Figure 3d) distinguish the contribution sources: while pristine FeTiO_3_ generates an intrinsic photocurrent, the incorporation of the Au network overlayer superimposes a distinct photothermal current component upon this baseline. This confirms the formation of the local thermal field and its promoting effect on charge separation. To reveal the correlation between microscopic current‐carrying properties and macroscopic electrical response, we measured the conductivity and carrier concentration of different samples and performed a comparative analysis, as shown in Figure 3e. Relative to pristine FeTiO_3_ and conventional Au‐loaded counterparts, the Au‐network engineered architecture demonstrates superior carrier mobility. This enhanced transport property effectively establishes high‐speed channels for charge migration, significantly facilitating the efficient diffusion and delivery of energetic carriers throughout the catalyst system. Impedance spectroscopy also confirms this view, as shown in Figure S17. KPFM characterized the Fermi level and electron transfer of the samples (Figure 3f and Figure S18). Compared with the Au standard sample, the Fermi level of FeTiO_3_ was found to be 4.67 eV (band structure diagrams are shown in Figures S19 and S20). Furthermore, the incorporation of the Au network overlayer substantially amplifies the density of photogenerated electrons. This accumulation manifests as a markedly increased contact potential difference (CPD) under illumination, signifying a strengthened built‐in electric field that effectively drives charge carrier separation. This result directly indicates that the optimized interfacial band structure and the increased built‐in electric field significantly enhanced the separation efficiency of photogenerated carriers and effectively suppressed recombination.

To delve into the ultrafast carrier dynamics of AuL‐FeTiO_3_ and verify its structural advantages, we used femtosecond transient absorption spectroscopy (fs‐TAs) to track the charge evolution process after photoexcitation (Figure 3g and Figure S21). AuL‐FeTiO_3_ exhibits a broad positive signal (ESA) within the detection range, with its duration extending to the nanosecond timescale. Through a tri‐exponential fitting, we resolved the dynamic process as follows: ultrafast cooling of hot electrons (τ_1_), migration of short‐lived carriers (τ_2_), and long‐lived carriers trapped by deep‐level defects (τ_3_) [30, 31, 32]. Here, τ_2_ represents the effective migration time of carriers between active sites, directly dominating the kinetic efficiency of CO_2_ reduction (Figure 3h), and is therefore a key indicator for evaluating interfacial charge transfer. These characteristics collectively indicate the existence of interfacial charge transfer in the AuL‐FeTiO_3_ system. Quantitative analysis of the fitting parameters (Table S7 and Figure 3i) reveals that the AuL‐FeTiO_3_ architecture exhibits a substantially prolonged τ_2_ lifetime. This enhancement is attributed to the dual benefits of suppressed non‐radiative recombination and an optimized interfacial electric field enabled by the Au network overlayer. Furthermore, we quantified the electron transfer rate (Δk_ET_, Figure 3j) [33]. The results show that, compared with the conventional Au‐loaded sample, the AuL overlayer structure significantly improves Δk_ET_. In summary, the Au network overlayer achieves dual kinetic optimization of extended lifetime (τ_2_) and accelerated transport (Δk_ET_). This mechanism ensures that photogenerated carriers not only “survive” longer but also separate and inject into reaction sites more efficiently, thus providing strong kinetic support for the photothermal reduction of CO_2_.

### 2.4 Adsorption and Activation

**FIGURE 4:**
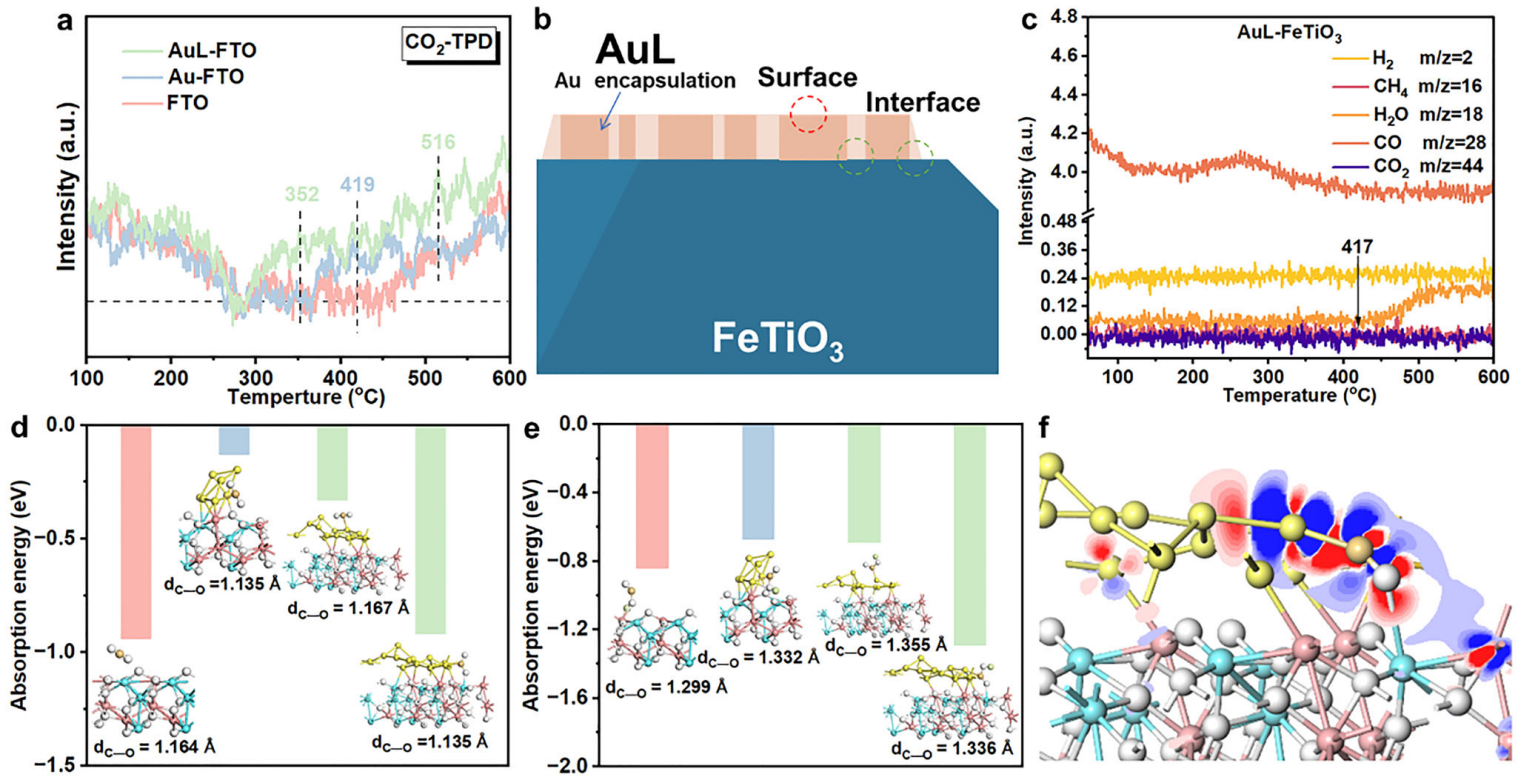
Adsorption. (a) Temperature‐programmed CO_2_ desorption diagram. (b) Schematic diagram of different adsorption sites of AuL‐overlayer (the red circle is the AuL surface, and the green circle is the AuL interface). (c) Temperature‐programmed surface reaction diagram of AuL‐FeTiO_3_. Adsorption of CO_2_ (d) and COOH (e) on different samples. (f) Charge density difference of CO adsorbed on AuL‐FeTiO_3_. (Blue electron cloud indicates surface electron accumulation, red indicates electron dissipation).

To systematically evaluate the effect of the Au network capping layer on reactant adsorption and activation, we conducted H_2_ temperature‐programmed desorption (H_2_‐TPD) experiments and compared the catalytic performance with that of FeTiO_3_ samples (Figure 4a). The introduction of Au generated new adsorption sites on Au‐FeTiO_3_ and AuL‐FeTiO_3_, as shown in Figure 4b. Furthermore, thanks to its unique network topology, the gold network architecture exposed a high density of accessible surface and interfacial adsorption sites. Temperature‐programmed surface reaction (TPSR) was used to characterize the reaction behavior of the catalyst surface at different temperatures, as shown in Figure 4c and Figure S22 [34, 35]. AuL‐FeTiO_3_ exhibits a distinct H_2_O desorption signal at 417°C, indicating that its active sites are more favorable for water desorption, allowing the sites to return to an available state more quickly for new reaction cycles. Furthermore, the stronger CO signal on AuL‐FeTiO_3_ supports its higher CO yield. DFT simulations were used to calculate the adsorption energies of reactants and intermediates to evaluate their adsorption behavior (Figure 4d,e and Figure S23). Compared to other samples, AuL‐FeTiO_3_ has a higher site diversity, allowing different adsorbed species to select the most suitable adsorption and activation sites. Adsorption energy and activation alone cannot reveal the migration of electrons between the adsorbate and the adsorption site, so the electronic origins of adsorption and activation are analyzed by differential charge density (Figure 4f and Figure S24). The amount of CO electron transfer at the AuL‐overlaid FeTiO_3_ interface is higher than that at Au‐FeTiO_3_, which means that AuL‐FeTiO_3_ has a higher concentration of surface electrons. In other words, the electronic structure at the AuL‐FeTiO_3_ interface is better than that at Au‐FeTiO_3_. In summary, AuL‐FeTiO_3_ has more adsorption sites and a better electronic structure at the interface and surface, which is conducive to the adsorption, migration, and activation of reactants.

### 2.5 Reaction Mechanism

**FIGURE 5:**
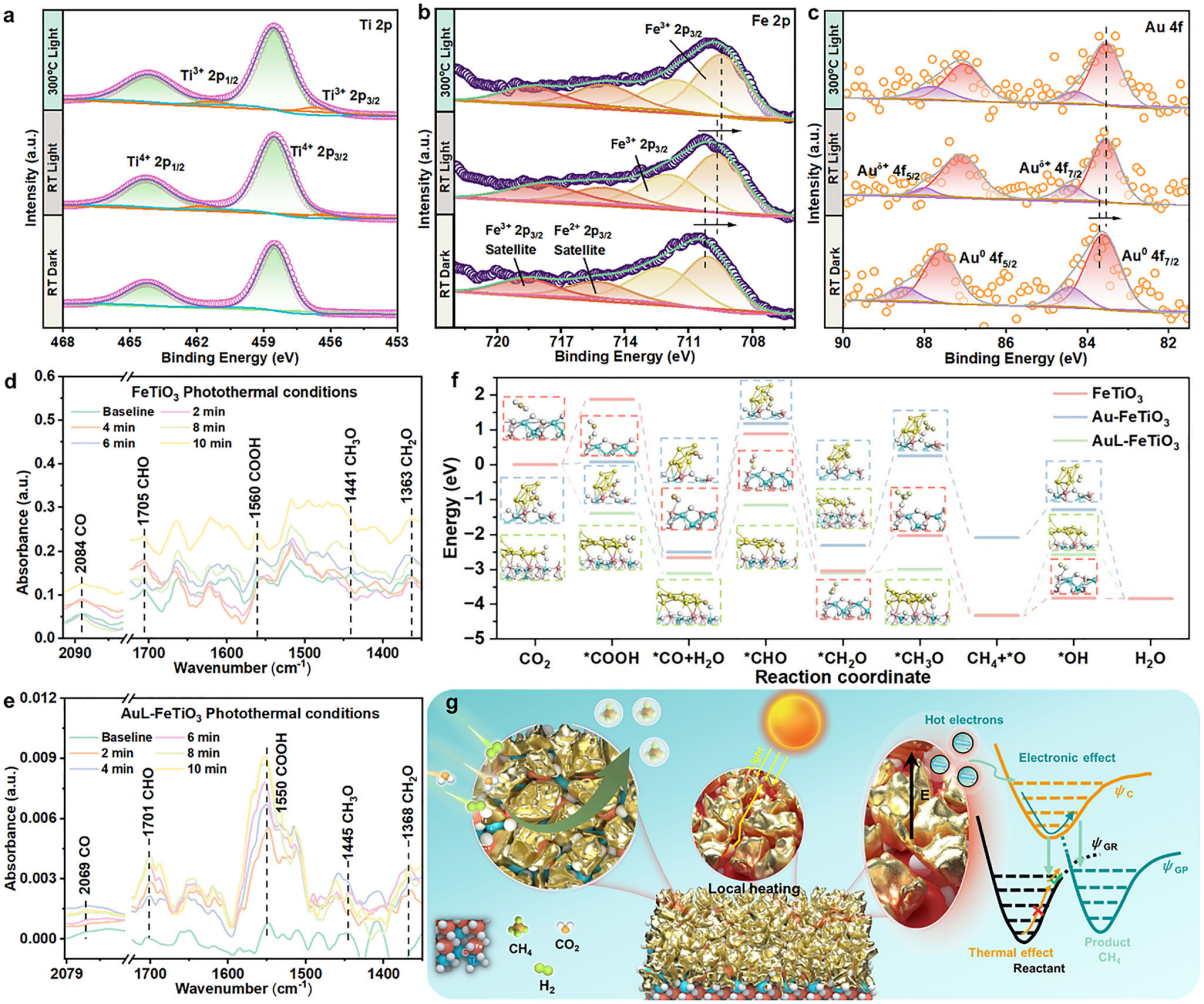
Reaction mechanism. In situ XPS spectrum of AuL‐FeTiO_3_, Au 4f (a), Ti 2p (b), and Fe 2p (c). In situ FTIR spectra of FeTiO_3_ (d) and AuL‐FeTiO_3_ (e) under photothermal conditions. (f) Reaction pathway diagram of the samples. (g) Reaction mechanism diagram.

In the AuL‐FeTiO_3_ photothermal catalytic system, we investigated carrier dynamics in depth using in situ XPS technology, successfully decoupling the specific contributions of the optical and thermal fields to electron behavior. The intimate interfacial contact established between the Au network overlayer and the FeTiO_3_ substrate necessitates Fermi level equilibration. This thermodynamic alignment drives a spontaneous electron transfer from FeTiO_3_ to Au, thereby establishing a robust built‐in electric field (Schottky barrier). The in situ XPS spectra clearly reveal the microscopic mechanism of this “photothermal division of labor”. Specifically, the binding energy of Au 4f shifts significantly to lower energies under illumination, but does not change significantly when a thermal field is subsequently introduced (Figure 5a), indicating that the electron enrichment on the Au surface mainly originates from the non‐thermal photoexcitation effect of LSPR. In stark contrast, while the Fe species in FeTiO_3_ exhibit only slight displacement under illumination, they display a significant “secondary shift mode” toward lower energies under thermal conditions (Figure 5b). This phenomenon confirms that electron accumulation at Fe sites is highly modulated by thermal effects—that is, enhanced lattice vibrations (phonon‐assisted) effectively lower the activation energy of polaron hopping, thereby accelerating electron transport and enrichment in the oxide lattice. The proportion of Ti^3^^+^ in Ti 2p and the proportion of oxygen vacancies in O 1s also support this conclusion (Figure 5c and Figure S25). Based on this, the operating mechanism of the system can be summarized as follows: the LSPR acts as an “electron pump” to generate hot electrons, while the localized thermal field acts as a “kinetic accelerator” to drive the efficient transport of electrons to the active sites (Fe^2^^+^/Ti^3^^+^). Finally, the observedor binding energy recovery upon the introduction of the CO_2_ and H_2_ reaction atmosphere serves as definitive proof that these accumulated energetic carriers—comprising both lattice electrons and LSPR‐derived hot electrons—are efficiently depleted during the CO_2_ reduction process. This consumption directly drives the adsorption and subsequent transformation of key intermediates, specifically *CO and *COOH.

According to literature reports, COOH and CH_x_O are key hydrogenation intermediates in several important pathways of CH_4_ formation [36, 37]. Their adsorption strength and presence determine whether the reaction can proceed. Through further analysis of in situ infrared spectroscopy and reaction pathways, we found that under photothermal conditions, no intermediate signals such as CH_x_O were observed in FeTiO_3_, indicating a lack of key factors for CH_4_ formation (Figure 5d). This may be due to the constraint of the rate‐determining step (RDS). The reaction pathway of the sample is shown in Figure 5f and Figure S27, with the RDS being *CO→*CHO. Au‐FeTiO_3_ under photothermal conditions and AuL‐FeTiO_3_ under both photo‐irradiation and photothermal conditions can detect this key intermediate, consistent with its performance (Figure S26). The AuL‐FeTiO_3_ structure exhibits a significant photothermal effect, generating a local thermal field, thereby increasing the surface temperature. This local heating can act as a kinetic promoter, effectively reducing the reaction activation energy Ea, thereby significantly increasing the yield of the reduction product.

## 3 Conclusion

In summary, this study proposes an innovative spectral‐kinetic synergistic strategy to successfully overcome the kinetic bottleneck of CO_2_ photothermal reduction by constructing a FeTiO_3_ catalyst modified with an Au network overlayer. The core advantage of this catalyst lies in its unique Au network overlayer topology: it not only constructs a high‐density Au‐FeTiO_3_ interfacial active site but also realizes a highly efficient multi‐field coupling system. Mechanistic studies show that the Au network generates high‐energy hot electrons through LSPR while efficiently capturing infrared photons to generate a localized thermal field. Crucially, through in situ characterization, we successfully decoupled the specific contributions of light and heat—revealing that the localized thermal field functions as a kinetic promoter. It significantly lowers the activation barrier for polaron transport, thereby driving the efficient injection and stabilization of LSPR‐derived hot electrons at the FeTiO_3_ active sites. This multi‐field coupling mechanism fundamentally optimizes the surface electronic structure and extends carrier lifetime, thereby achieving efficient and stable CO_2_ conversion under mild or even low‐temperature conditions. This study not only elucidates the microscopic mechanism of AuL overlayer in regulating electron/phonon behavior, but also provides a universal theoretical model and design blueprint for designing ISRU materials adapted to the extreme lunar environment.

## Author Contributions

**Yahang Wang**: investigation, writing – review and editing, data curation. **Quanxin Wang**: investigation, methodology. **Pakkin Leong**: supervision, resources, methodology. **Shi Feng**: software, formal analysis, data curation. **Meng Wang**: data curation, formal analysis. **Runyang Mo**: funding acquisition, resources, supervision. **Xianjin Shi**: investigation, validation, resources. **Xiaohui Li**: resources, funding acquisition, investigation. **Gangqiang Zhu**: supervision, resources, project administration. **Chipui Tang**: funding acquisition, supervision, resources, project administration.

## Conflicts of Interest

The authors declare no conflicts of interest.

## Supporting information




**Supporting File**: advs76857‐sup‐0001‐SuppMat.docx.

## Data Availability

The data that support the findings of this study are available from the corresponding author upon reasonable request.
