# Supplementary material for: Spectral‐Kinetic Synergy in Au‐Network Engineered FeTiO3: A Multi‐Field Coupling Strategy for Lunar In Situ Resource Utilization
Source: Adv Sci (Weinh). 2026 Aug 6:e76857. Online ahead of print. doi: 10.1002/advs.76857 (PMC13445315; doi:10.1002/advs.76857)
Supplement: Supplementary file 1 [file ADVS-9999-e76857-s001.docx]

**Supporting Information**

**Lunar in-situ resource utilization strategy: Multi-field coupling effect of gold-encapsulated FeTiO_3_ enables infrared light utilization and long electron lifetime**

Yahang Wang,^a^ Quanxin Wang,^b^ Pakkin Leong,^a^ Shi Feng,^a^ Meng Wang,^b^ Runyang Mo,^b^ Xianjin Shi,^c^ Xiaohui Li,^b^ Gangqiang Zhu^b^ and Chipui Tang^a,d^*

*^a^* *State Key Laboratory of Lunar and Planetary Sciences,* *Macau University of Science and Technology,* *Taipa 999078, Macao, P. R. China*

*^b^ School of Physics and Information Technology, Shaanxi Normal University, Xi’an 710062,* *P. R. China*

*^c^ State Key Lab of Loess and Quaternary Geology (SKLLQG), Institute of Earth Environment, Chinese Academy of Sciences, Xi’an 710061, P. R. China*

*^d^ Faculty of Innovation Engineering, Macau University of Science and Technology, Taipa 999078, Macao, P. R. China;*

*Corresponding author: Tel/Fax: +853-88971923;

Email address: [*cptang@must.edu.mo*](mailto:cptang@must.edu.mo)

**Supplementary Note 1. XRD, TEM and other catalyst characterization**

Crystal structures of all kinds of sample catalysts were analyzed with the X-ray diffraction (XRD) technique (Model D8 Advance, Bruker, Germany) with graphite high-intensity Cu Kα (λ = 0.15418 nm) radiation. XRD spectra were taken for 2θ angles from 10^o^- 80^o^ at a scan rate of 0.02^o^/min. Accelerating voltage and electric current were 40 kV and 40 mA, respectively.

The Brunauer Emmett Teller (BET) method was used for the specific surface area of the samples, and the Barrett Joyner Halenda (BJH) method was used for the pore diameter. N_2_ adsorption and desorption, BET and pore size were tested using BSD-660M from BSD Instrument Technology (Beijing) Co., The X-ray photoelectron spectroscopy (XPS) of the sample was performed by Escalab MkII spectrometer (VG Scienta, Node Materials: Al Kα, HV=1486.6 eV).

**Supplementary Note 2. Atomic force microscopy and work function measurement**

Ltd. Atomic force microscopy (KPFM) tests were performed using AtomExplorer from Truth Instruments Co., Ltd. The probe model was: HQ: NSC18/Pt, and the measurement range was 5 μm×5 μm. For measuring the work function of a sample, the work function is first calibrated using Au, and then the difference between the work function of the sample and Au is measured to indirectly measure the work function of the sample.

**Supplementary Note 3. In-situ FTIR** **test**

The sample was placed in a small crucible and then placed in an in-situ infrared testing chamber. The temperature was raised to 120°C under a nitrogen atmosphere and heat-treated for 30 minutes. After cooling to room temperature, measurements were taken under dark conditions at room temperature, light conditions at room temperature, and light conditions at 300°C. The gas mixture was 5% CO_2_ + H_2_, with the remaining 5% balanced with N_2_.

**Supplementary Note 4. Temperature Programmed Desorption (TPD) tests**

The TPD test of the catalyst was conducted on the catalyst dynamic characterization system. The specific steps of the test are as follows:

(1) Preparation stage: Place quartz wool at the bottom of the thicker section of the U-shaped tube, place 50 mg of catalyst on the quartz wool, and install the quartz tube into the catalyst dynamic characterization system;

(2) Pretreatment stage: Under argon purge, set the pretreatment temperature to 150^o^C, the heating rate to 10^o^C/min, and the pretreatment time to 2 h. After the treatment, wait for the temperature to drop to 50^o^C, purge with test gas (H_2_) for 60 min, and wait for baseline equilibrium. After the purge, cool down to room temperature to prepare for the next stage of the test;

(3) Test stage: The purge gas remains unchanged or is changed to argon, set the test temperature to 700 ^o^C, the heating rate to 10 ^o^C/min, and the test ends when the temperature rises to 700 ^o^C. During the test, the gas passing through the catalyst is monitored by a thermal conductivity detector (TCD).

**Supplementary Note 5. X-ray Absorption Fine Structure (XAFS) test**

X-ray absorption fine structure (XAFS) spectroscopy data of the Ti element were collected from the BL07U beamline of the Shanghai Synchrotron Radiation Facility, using the TEY mode. XAFS of the Fe sample was collected from the following equipment. The test specimens were prepared by the KBr-disk method. XAFS spectroscopy was carried out using the Rapid-XAFS1M (Anhui Ab-sorption Spectroscopy Analysis Instrument Co., Ltd,) by transmission mode at 25 kV and 40 mA.

**Supplementary Note 6. Evaluation of Catalyst CO_2_ Hydrogenation Performance**

Take 50 mg of catalyst sample (0.5 g for natural catalysts such as ilmenite) and place it in a quartz tube. Then place the quartz tube in a photothermal reaction device. The catalyst undergoes CO_2_ hydrogenation reaction under the conditions of 0.1 MPa pressure, 1:1 H_2_/CO_2_ ratio, and 12000 mL·g^-1^·h^-1^ space velocity. The temperature rise interval is 50^o^C, and 3 points are taken at each temperature point from room temperature to 300^o^C. At the same time, photothermal and thermal conditions are tested intermittently. For the catalyst selection process, the test is directly carried out under the conditions of 300^o^C plus light.

The selectivity of CO is calculated using formula (1), where the total yield is the sum of all products.

$CO selectivity=\frac{CO yield}{Total yield}$	(1)

**Supplementary Note 7. Catalyst performance evaluation at different wavelengths**

The performance of the catalyst was tested at 300°C by adding bandpass filters of 380 nm, 400 nm, 475 nm, 550 nm, 650 nm and 700 nm to the xenon lamp. The 800 nm test used a cutoff filter to filter out light below 800 nm.

**Supplementary Note 8. DFT calculation details**

We used the first-principles calculation method based on density functional theory (DFT) and used the Vienna ab initio simulation package (VASP) for calculations. The interaction between ions and electrons was described by the augmented wave (PAW) method, and the exchange correlation was described by the generalized gradient approximation (GGA) of the Perdew-Burke Enzerhor (PBE) functional. Both the FeTiO_3_ surface and the AuL-FeTiO_3_ surface were calculated using a 3×3×1 supercell. The cutoff energy was set to 400 eV. A 20 Å vacuum layer was set to avoid periodic repetition effects. The Brillouin zone was sampled with a 1×1×1 k-point grid, centered on the geometry optimization. The energy convergence criterion and mechanical convergence criterion for all structural optimizations were set to 1.0 × 10^-4^ eV/atom and 0.01 eV/Å, respectively.

The adsorption energy (E_ads_) is calculated as follows:

E_ads_ = E_total_ – E_surface_ – E_adsorbates_

Where E_total_, E_surface_ and E_adsorbates_ are the energy of substrate and adsorbate, respectively.

The change in the Gibbs free energy (∆G) value of each step in the reduction process is calculated by correcting the zero-point energy and entropy of DFT. The calculation formula is as follows:

∆G=∆E + ∆ZPE - T∆S

∆E is obtained by DFT calculation, ∆ZPE is the zero-point energy, T is 298.15 K, and ∆S is the entropy change.

**Supplementary Figure 1.** FTIR spectra of FeTiO_3_, Au-FeTiO_3_ and AuL-FeTiO_3_.

**Supplementary Figure 2.** Pore size, N_2_ adsorption-desorption curves and specific surface area of FeTiO_3_(a), Au-FeTiO_3_(b), and AuL-FeTiO_3_(c).

**Supplementary Figure 3.** High-resolution XPS spectra of Ti 2p(a), Fe 2p(b), O 1s(c) Au 3d(d), and full spectrum(e).

**Supplementary Figure 4.** Original data and fitted data of the imaginary part (a), real part (b) and K space (c) of the Fe element in AuL-FeTiO_3_.

**Supplementary Figure 5.** Original data and fitted data of the imaginary part, real part and K space of the Ti element in FeTiO_3_ (a, b, c) and AuL-FeTiO3 (d, e, f).

**Supplementary Figure 6.** Wavelet transforms of Fe-foil (a), Fe_3_O_4_ (b), and FeO (c).

**Supplementary Figure 7.** SEM image of AuL-FeTiO_3_.

**Supplementary Figure 8.** TEM image and mapping of Au-FeTiO_3_.

**Supplementary Figure 9.**TEM image of AuL-FeTiO_3_

**Supplementary Figure 10.** HAADF-STEM (a, d) and BF-STEM (b, e) images of AuL. (c, f) Energy Loss Spectrum of AuL-FeTiO₃.

**Supplementary Figure 11.** Infrared thermal images of FeTiO₃ (a, b) and Au-FeTiO₃ (c, d) taken at different wavelengths.

**Supplementary Figure 12.** Tacu diagrams of FeTiO₃, Au-FeTiO₃ and AuL-FeTiO₃

**Supplementary Figure 13.** (a) TEM image of Au-FeTiO_3_, (b) SEM image of AuL-FeTiO_3_, (c) TEM image of AuL-FeTiO3. (d-f) FDTD simulation diagrams of Au nanostructures on Au-FeTiO_3_ and AuL-FeTiO_3_ after analysis by SEM and TEM. LSPR simulations of near-field electric field intensity of Au nanoparticles (g), Au nanorods (h), and AuL structures (l).

The evolution of our understanding of the microscopic topology of AuL closely aligns with the iterative process of FDTD theoretical modeling. First, SEM characterization revealed a distinct mesh-like topology on the AuL surface; however, limited by low magnification resolution, the specific microstructural building blocks could not be directly analyzed at this stage. Based on this mesh-like geometric profile, we initially hypothesized that the network might consist of interwoven nanorods and established a preliminary FDTD simulation model (Figure e). Subsequently, TEM was used to perform a more refined microstructural analysis of AuL, confirming that the mesh-like topology is actually composed of a large number of tightly packed Au nanoparticles. Based on this realistic physical morphology, we revised and improved the initial model, ultimately constructing a model that better fits the AuL topology (Figure f).

**Supplementary Figure 14.** Photothermal CO₂ hydrogenation performance of FeTiO₃ (a) and Au-FeTiO₃ (b) and their selectivity for CO (test conditions: 0-300℃, 15 mW·cm⁻²).

**Supplementary Figure 15.** Stability test of AuL-FeTiO_3_.

**Supplementary Figure 16.** High-resolution XPS spectra of Ti 2p(a), Fe 2p(b), O 1s(c) Au 3d(d), and full spectrum(e).

**Supplementary Figure 17.** Impedance spectrum of FeTiO₃, Au-FeTiO₃ and AuL-FeTiO₃.

**Supplementary Figure 18.** KPFM plots of Au standard and FeTiO₃ and AuL-FeTiO₃.

**Supplementary Figure 19.** The Fermi level of FeTiO₃.

**Supplementary Figure 20.** Fermi level changes of FeTiO₃ and AuL-FeTiO₃.

**Supplementary Figure 21.** fs-TAs spectra of FeTiO₃ and Au-FeTiO₃.

**Supplementary Figure 22.** Temperature-programmed surface reaction diagrams of FeTiO₃ and Au-FeTiO₃.

**Supplementary Figure 23.** Adsorption energy, bond length and bond angle of the sample for CO_2_, CO, H_2_ and COOH.

**Supplementary Figure 24.** Differential charge density of Au-FeTiO₃ for CO adsorption.

**Supplementary Figure 25.** In-situ XPS full spectrum (a) and high-resolution O 1s (b).

**Supplementary Figure 26.** In-situ FTIR of Au-FeTiO_3_ and AuL-FeTiO_3_ under different reaction conditions.

It is noteworthy that although in-situ infrared spectroscopy could clearly capture signals of key intermediates such as *COOH and *CH_x_O under purely thermal conditions, no CH_4_ formation was detected in macroscopic catalytic tests. This seemingly contradictory phenomenon profoundly reveals the unique mechanism of multi-field coupled catalysis. Under purely thermal conditions, AuL-FeTiO_3_, through its regulated interfacial structure, successfully lowered the energy barrier of RDS to 1.95 eV, thereby thermodynamically driving the formation and surface accumulation of initial hydrogenated species. However, DFT calculations show that the reaction encountered a secondary kinetic deadlock in the subsequent deep hydrogenation and desorption stages—its key steps (such as the CH_4_ + *O → *OH) faced a high energy barrier as high as 1.73 eV. Under purely thermochemical driving, simple lattice thermal vibrations simply cannot overcome such a huge secondary energy barrier, causing these intermediates to be trapped in the "thermodynamic valley" of the surface and unable to be converted into macroscopic gaseous products. Only under the combined action of photothermal multi-field coupling can the high-energy hot electron injection induced by the AuL topology and the localized enhanced electric field effectively activate these "trapped" intermediates, forcibly cross the high potential barrier of 1.73 eV, and finally open up the last kilometer to CH_4_.

**Supplementary Figure 27.** Reaction pathway model.

**Supplementary** **Table 1.** Ea activation energy fitting data

|  | A | k | Ea (kJ/mol) |
| --- | --- | --- | --- |
| FeTiO_3_  Photo-thermal | 7.51 | 5.02 | 41.74 |
| Au-FeTiO_3_  Photo-thermal | 10.47 | 2.62 | 21.78 |
| AuL-FeTiO_3_  Thermal | 12.50 | 4.35 | 36.16 |
| AuL-FeTiO_3_  Photo-thermal | 11.30 | 1.80 | 14.97 |

**Supplementary** **Table 2.** Transient fluorescence biexponential fitting data

|  | τ_1_ | A1 | τ_2_ | A2 | <τ> |
| --- | --- | --- | --- | --- | --- |
| FeTiO_3_ | 1.29 | 0.64 | 4.25 | 0.29 | 3.05 |
| Au-FeTiO_3_ | 1.53 | 0.81 | 5.76 | 0.11 | 2.99 |
| AuL-FeTiO_3_ | 0.79 | 0.74 | 4.03 | 0.23 | 2.77 |

**Supplementary** **Table 3.** X-ray Photoelectron Spectroscopy Fitting Data

| Samples | Molar ratio (%) | | | O_latt_ (%) | O_vac_ (%) |
| --- | --- | --- | --- | --- | --- |
|  | Au^δ+^/Au_total_ | Ti^4+^/Ti_total_ | Fe^2+^/Fe_total_ |  |  |
| FeTiO_3_ | — | 100 | 53.5 | 84.7 | 15.3 |
| Au-FeTiO_3_ | 0 | 100 | 54.1 | 83.4 | 16.6 |
| AuL-FeTiO_3_ | 9.9 | 100 | 53.5 | 78.7 | 21.3 |
| AuL-FeTiO_3_  after | 13.0 | 100 | 53.5 | 78.1 | 21.9 |

**Supplementary** **Table 4.** In-situ XPS fitting data of AuL-FeTiO_3_

| Samples | Molar ratio (%) | | | O_latt_ (%) | O_vac_ (%) |
| --- | --- | --- | --- | --- | --- |
|  | Au^δ+^/Au_total_ | Ti^4+^/Ti_total_ | Fe^2+^/Fe_total_ |  |  |
| Dark | 18.7 | 100 | 53.9 | 86.2 | 13.8 |
| Light | 17.4 | 95.2 | 60.2 | 86.2 | 13.8 |
| Light+300 ^o^C | 14. | 90.1 | 62.5 | 84.7 | 15.3 |
| Light+300 ^o^C  ＆CO_2_+H_2_ | 17.4 | 87.7 | 61.0 | 88.5 | 11.5 |

**Supplementary** **Table 5.** Fitting parameters of the Ti-refined EXAFS spectra of samples

| **Sample** | **Path** | **N** | **R (Å)** | **ΔE_0_ (eV)** | **σ^2^ (Å^2^)** | **R-factor** |
| --- | --- | --- | --- | --- | --- | --- |
| Ti foil | Ti–Ti | 8.0 | 2.90 | 4.99 | 0.007 | 0.014 |
| TiO_2_ | Ti–O | 2.0 | 1.93 | -6.48 | 0.009 | 0.013 |
|  | Ti–O–Ti | 4.0 | 3.69 |  | 0.017 |  |
| FeTiO_3_ | Ti–O | 3.0 | 1.82 | -9.16 | 0.003 | 0.022 |
|  | Ti–O–Fe | 2.0 | 2.60 |  | 0.002 |  |
| AuL-FeTiO_3_ | Ti–O | 3.0 | 2.05 | -8.47 | 0.010 | 0.032 |
|  | Ti–O–Fe | 2.0 | 3.15 |  | 0.014 |  |

(N: coordination number; R: distance; E_0_: energy shift; σ^2^: mean-square disorder; R-factor: Errors in data fitting).

**Supplementary** **Table 6.** Fitting parameters of the Fe-refined EXAFS spectra of samples

| **Sample** | **Path** | **N** | **R (Å)** | **ΔE_0_ (eV)** | **σ^2^ (Å^2^)** | **R-factor** |
| --- | --- | --- | --- | --- | --- | --- |
| Fe foil | Fe–Fe | 8.0 | 2.44 | 2.64 | 0.006 | 0.014 |
| FeO | Fe–O | 6.0 | 2.11 | -3.26 | 0.013 | 0.010 |
|  | Fe–O–Fe | 12.0 | 3.07 |  | 0.013 |  |
| Fe_2_O_3_ | Fe–O | 4.0 | 1.96 | -7.28 | 0.013 | 0.012 |
|  | Fe–O–Fe | 4.0 | 3.00 |  | 0.009 |  |
| Fe_3_O_4_ | Fe–O | 3.0 | 1.99 | -6.75 | 0.017 | 0.008 |
|  | Fe–O–Fe | 2.0 | 3.00 |  | 0.009 |  |
| FeTiO_3_ | Fe–O | 3.0 | 1.92 | -8.40 | 0.016 | 0.025 |
|  | Fe–O–Ti | 1.0 | 2.67 |  | 0.009 |  |
| AuL-FeTiO_3_ | Fe–O | 3.0 | 2.00 | -1.12 | 0.007 | 0.006 |
|  | Fe–O–Ti | 2.0 | 2.96 |  | 0.001 |  |

(N: coordination number; R: distance; E_0_: energy shift; σ^2^: mean-square disorder; R-factor: Errors in data fitting).

**Supplementary** **Table 7.** Transient absorption electron dynamics fitting data

| Sample | τ_1_ (ps) | A1 | τ_2_ (ps) | A2 | τ_3_ (ps) | A3 |
| --- | --- | --- | --- | --- | --- | --- |
| FeTiO_3_ | 0.8 | 0.026 | 23.4 | 4.9×10^-4^ | 566 | 3.9×10^-4^ |
| Au-FeTiO_3_ | 1.7 | 0.002 | 32.9 | 2.3×10^-4^ | 603 | 1.5×10^-4^ |
| AuL-FeTiO_3_ | 1.1 | 0.010 | 39.5 | 7.2×10^-4^ | 558.3 | 5.3×10^-4^ |
